# Short-Term Effect of Nutrient Availability and Rainfall Distribution on Biomass Production and Leaf Nutrient Content of Savanna Tree Species

**DOI:** 10.1371/journal.pone.0092619

**Published:** 2014-03-25

**Authors:** Eduardo R. M. Barbosa, Kyle W. Tomlinson, Luísa G. Carvalheiro, Kevin Kirkman, Steven de Bie, Herbert H. T. Prins, Frank van Langevelde

**Affiliations:** 1 Resource Ecology Group, Wageningen University, Wageningen, The Netherlands; 2 Departamento de Botânica, Laboratório de Termobiologia, Instituto de Ciências Biológicas, Universidade de Brasília, Brasília, DF, Brazil; 3 Community Ecology & Conservation Group, Xishuangbanna Tropical Botanical Garden, Chinese Academy of Sciences, Yunnan, China; 4 School of Biology, University of Leeds, Leeds, the United Kingdom; 5 Terestrial Zoology, Naturalis Biodiversity Center, Leiden, The Netherlands; 6 School of Life Sciences, University of KwaZulu-Natal, Scottsville, South Africa; DOE Pacific Northwest National Laboratory, United States of America

## Abstract

Changes in land use may lead to increased soil nutrient levels in many ecosystems (e.g. due to intensification of agricultural fertilizer use). Plant species differ widely in their response to differences in soil nutrients, and for savannas it is uncertain how this nutrient enrichment will affect plant community dynamics. We set up a large controlled short-term experiment in a semi-arid savanna to test how water supply (even water supply vs. natural rainfall) and nutrient availability (no fertilisation vs. fertilisation) affects seedlings’ above-ground biomass production and leaf-nutrient concentrations (N, P and K) of broad-leafed and fine-leafed tree species. Contrary to expectations, neither changes in water supply nor changes in soil nutrient level affected biomass production of the studied species. By contrast, leaf-nutrient concentration did change significantly. Under regular water supply, soil nutrient addition increased the leaf phosphorus concentration of both fine-leafed and broad-leafed species. However, under uneven water supply, leaf nitrogen and phosphorus concentration declined with soil nutrient supply, this effect being more accentuated in broad-leafed species. Leaf potassium concentration of broad-leafed species was lower when growing under constant water supply, especially when no NPK fertilizer was applied. We found that changes in environmental factors can affect leaf quality, indicating a potential interactive effect between land-use changes and environmental changes on savanna vegetation: under more uneven rainfall patterns within the growing season, leaf quality of tree seedlings for a number of species can change as a response to changes in nutrient levels, even if overall plant biomass does not change. Such changes might affect herbivore pressure on trees and thus savanna plant community dynamics. Although longer term experiments would be essential to test such potential effects of eutrophication via changes in leaf nutrient concentration, our findings provide important insights that can help guide management plans that aim to preserve savanna biodiversity.

## Introduction

Recent studies predict an increase in nitrogen deposition over southern Africa during the next few decades [1], due to rising industrial emissions and changes in land use [2]. Soil nitrogen enrichment can lead to soil acidification, which reduces soil fertility by promoting leaching of certain nutrients (such as calcium and magnesium) [3]. Moreover, increased nitrogen availability might also affect the carbon flux from soils of natural ecosystems [4] through changes in plant and soil microbial communities [5]. Such environmental changes can have important impacts for African savannas, especially on the species composition and abundance. Furthermore, alterations in rainfall patterns are also expected in the region where savannas occur [6]. However, little information on the effects of changes in soil nutrient and water availability on the leaf nutrient concentration of savanna trees is found in the literature [7].

Plant productivity and above-ground biomass are thought to increase with higher soil resource availability (e.g. nitrogen, water, phosphorus) [8]–[10]. In drier regions (such as semi-arid savannas), highly variable rainfall may negatively affect plant nutrient uptake and storage [11], [12], potentially limiting plant growth during the growing season [13]. Indeed, performance of savanna tree seedlings is suggested to be worse when grown in nutrient-rich soils than in nutrient-poor soils [14], [15]. This effect may be caused by the intensification of herbaceous competition for water and not by direct negative effects of high nutrient availability on tree seedlings [15]. Moreover, increased amounts of nutrients in plant leaves might increase their quality as food for herbivores [16]–[18], whereas increased water availability may increase biomass but decrease leaf nutrient concentration [18]. Tree seedling recruitment is a critical stage in the regeneration of trees and overall plant population dynamics [19]–[22]. However, there is a lack of empirical studies involving multiple plant species [22]. Most experimental studies evaluating the growth of tree species in response to resource supply and disturbance, with and without grass competitors, focus on single species [23], [24], and there are few comparative investigations on seedlings of savanna tree species either within or across communities. This lack of empirical knowledge critically limits our ability to understand how seedlings of different species in a community perform under different environmental condition, and consequently, how plant community dynamics might change under modifications in the land use and climate conditions [25], [26].

As plant species differ widely in their response to differences in soil nutrients [27], changes in soil resource availability (water and nutrient availability) may change structural heterogeneity in tree cover [28] or leaf nutrient concentration, and thereby influence primary productivity [29], [30]. Differences in functional traits can mechanistically explain why species differ in their performance across resource and disturbance gradients [31], [32]. Qualitative trait differences between species which are associated with nutrient and water gradients have been recognised [33]. Notably, within African savannas, dystrophic or humid savannas are dominated by broad-leafed species that are also non-spinescent, non-N-fixing species, whereas eutrophic or arid savannas are dominated by fine-leafed species which may additionally be spinescent or N-fixing [33]–[36]. These two groups can also be distinguished on the basis of their leaf chemistry, physiology and morphology [32]. As these functional traits are already found in tree seedlings during their first season of growth (e.g. N-fixing associations can be established early as two/three weeks after planting) [37], there is reason to believe that seedlings of tree species representing these functional species groups respond differently to changes in supply rates of resources, and that these differences may in part explain why they dominate in different environments.

Savannas are often characterized by water-limited plant growth during the growing season [38]. However, the amount of rainfall within the wet season is highly unpredictable, especially in semi-arid savannas [39]. Dry periods during the wet season can have an important impact limiting tree seedling survival [28]. Such dry periods may become more frequent in the near future, as global climate models indicate rising temperatures and increasingly erratic rainfall patterns across Southern African regions [40], [41]. Climatic changes may also lead to slightly extended later summer rainfall over eastern South Africa [42]. Here we evaluated the short-term effects of water variability (even water supply vs. natural rainfall) and soil nutrient availability (no fertilisation vs. NPK addition) on above-ground biomass production and leaf nutrient concentrations of seedlings of two important functional groups of semi-arid savanna trees: broad-leafed (4 species) and fine-leafed (4 species) species (Table 1). We focus on the leaf concentrations of nitrogen (N), phosphorus (P) and potassium (K) because these nutrients are important in many plant metabolism processes [43], and in the diets of herbivores [16], [18].

**Table 1:** Functional trait data for tree species used in the experiment.

Species	Family	Sub-family	N_2_-fixing‡	Leaf type‡‡	Leaf size (cm^2^)††	Spinescence
**Fine-leafed species**						
*Acacia nigrescens* Oliv.	Fabaceae	Mimosoideae	Yes	Bipinnate	16.0 (±3.6)	Yes
*Acacia nilotica* Willd.	Fabaceae	Mimosoideae	Yes	Bipinnate	12.0 (±2.1)	Yes
*Acacia tortilis* Hayne	Fabaceae	Mimosoideae	Yes	Bipinnate	12.5 (±7.0)	Yes
*Dichrostachys cinerea* Wight and Arn	Fabaceae	Mimosoideae	Yes	Bipinnate	31.7 (±28.4)	Yes
**Broad-leafed species**						
*Colophospermum mopane* J. Léonard	Fabaceae	Caesalpinioideae	No	Pinnate	47.2 (±21.6)	No
*Combretum apiculatum* Sond.	Combretaceae	–	No	Simple	25.3 (±5.3)	No
*Schotia brachypetala* Sond.	Fabaceae	Caesalpinioideae	No	Pinnate	42.6 (±17.6)	No
*Peltophorum africanum* Sond.	Fabaceae	Caesalpinioideae	No	Bipinnate	99.5 (±81.8)	No

For continuous values the standard deviation is indicated between brackets. Sources of data are indicated in postscripts.

‡ [51], [70].

†† Obtained from the experimental seedlings from the treatment W0N0 (Natural rainfall-No nutrient addition).

‡‡ [70].

As increases in soil resources and reduction of periods of soil moisture deficiency are thought to increase plant productivity [8]–[11], we expected that all species respond positively to even water availability (no dry periods during the wet season) and to increased nutrient supply by increasing above-ground plant biomass (Hypothesis 1). As fine-leafed species are the dominant tree species in nutrient-rich savannas [35], are N-fixing and may have greater photosynthetic rates [44] than broad-leafed species, we expected fine-leafed species to have always higher leaf nutrient concentrations than broad-leafed species (Hypothesis 2). However, during growth, most nutrients (50–75%) are thought to be located in the leaves (e.g. [45], [46], their concentration depending mostly on soil nutrient availability [47], [48] and soil moisture [49]. Longer periods of soil moisture availability during the growing season may decrease leaf nutrient concentration, due to dilution effects of increased plant growth [18], [50]. Therefore, we expected that the two species groups would increase leaf nutrient concentration with increasing soil nutrient availability, and that it would decrease with constant water supply (Hypothesis 3).

## Methods

To test whether the two functional species groups differed in their response to variation in the growth conditions, we set up a large controlled, short-term field experiment in the Lowveld savanna region [35]. The study was carried out on private land of the Southern African Wildlife College (SAWC), Limpopo Province, South Africa (24°15′20.23″S, 31°23′23.63″E). For future permissions for fieldwork at the SAWC please contact Mrs. Theresa Sowry (tsowry@sawc.org.za) or Mr. Francois Nel (fnel@sawc.org.za). The experiments were run during the wet season of 2009–2010 (November–May), in a fenced area that excluded large herbivores. The mean rainfall during the growing season (from October till April) of the previous 10 years (2000–2010) is *ca.* 456 mm (Satara Camp, Kruger Park around 40 km northeast of the research site). The mean maximum temperature during January (hottest month) is 33.7°C and the mean minimum temperature for June (coolest month) is 9.4°C [42]. The vegetation is described as Granite Lowveld [35], and the area is classified as semi-arid under the Köppen-Geiger System [36]. Soils in the experimental site were shallow (ca. 1.5 m depth) and mainly derived from granite [34] with occasional gabbro extrusions. Soils derived from granite tend to be coarse-textured and nutrient-poor (i.e., low availability of N and P) on crests and mid-slopes [51], but nutrient availability may be elevated in bottom positions in the landscape, and very locally such as on termitaria or underneath large *Acacia* trees [51].

### Species

We selected eight locally abundant tree species that make up a large proportion of vegetation cover in the Lowveld savanna region in South Africa. Although most of the selected species belong to the Fabaceae family (with the exception of *Combretum apiculatum*), these species are classified into two different sub-families: Mimosoideae (fine-leafed species) and Caesalpinioideae (broad-leafed species). In African semi-arid savannas, broad-leafed and medium-leafed species are found on dystrophic soils, characterised by high fire frequency (annual to triennial) and MAPs from 600–1500 mm [14]. Fine-leafed species (largely Mimosoideae) are found on eutrophic soils or skeletal soils with low fire frequency (quintennial or longer) and MAPs of 300–800 mm [35]. The study species were separated in two different functional species groups: four species with characteristic small leaves, spines, and N-fixing associations (hereafter termed ‘fine-leafed species’), and four species with characteristic broad leaves, no spines, and lacking N-fixing associations (hereafter termed ‘broad-leafed species’) (see Table 1). All the seeds used in the experiment were collected in areas surrounding the experimental site. Since these species are abundant in the savannas of Southern African region, changes in their populations due to varying environmental conditions will likely have substantial effects on the local vegetation structure.

### Treatments

**Figure 1:**
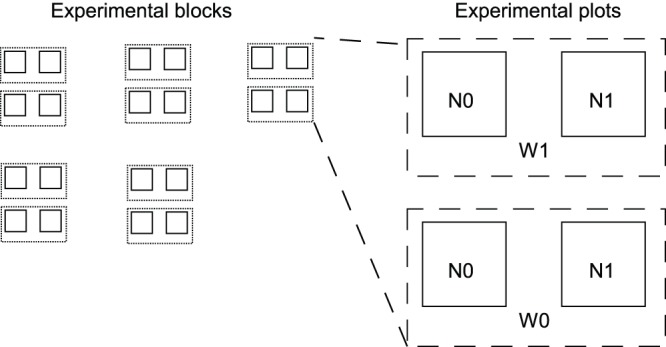
The experimental design. W0 - natural rainfall treatment, W1– even watering treatment, N0– no addition of nitrogen-phosphorus-potassium fertilizer, N1 addition of nitrogen-phosphorus-potassium fertilizer.

The study site was ploughed (about 20 cm deep) to homogenize the soil and to give all treatments the same starting conditions. Five blocks were laid out in a restricted area (90×90 m) in the experimental area (maximum distance between the blocks was 40 m). Inside each block, four 4-m^2^ plots were located, separated by a 2 m gap between the plots (Figure 1) Seedlings were subjected to two different water regimes: one covered with a rain-out shelter (W1 - even water supply) and another exposed to natural rainfall conditions (W0 - natural rainfall or uneven water supply). The nutrient treatment was separated in two different nutrient applications (N0 - no nutrient supply vs. N1 - nutrient supply), leading to a total of 20 experimental plots.

Three weeks before the experiment all seeds were sown in nursery bags (using the same soil of the experimental area). At four weeks after germination, 20 seedlings per species were randomly transplanted in treatment combination plots (five replicate blocks, each with four seedlings per plot). The seedling positions in the plots were randomly selected. The seedling density inside of the plots (20 seedlings per m^2^) was lower than the normal early seedling density in savannas (more than 50 seedlings per m^2^ in the seed/seedling bank [52]. The seedlings were then followed for six months (November 2009 to May 2010). Although our experiments were performed during a short period of time (six months), this period of time is equivalent to a growing season in the area where the study was conducted. As savanna tree species show great differences in growth strategies, which allow them to cope with the high unpredictability of the amount of annual rainfall [39], we expected differences in the responses to the variation in resource avaliabiity between species even in short-term experiments.

**Figure 2:**
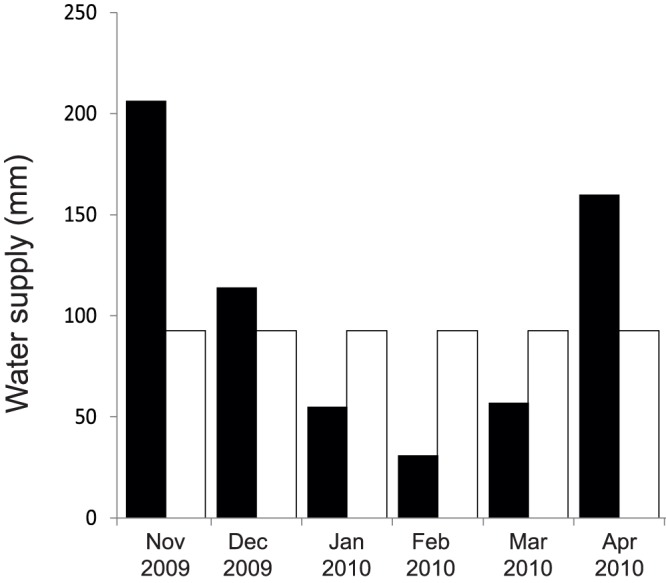
Monthly water availability (mm) during the experimental period. The black bars represent the monthly rainfall in the area during the wet season of 2009–2010 (natural rainfall treatment-W0). The white bars represent the monthly water supplied in the treatment W1. The rainfall data are from Satara Camp, Kruger Park around weather station (40 km northeast from the research site).

Plots within the uneven water supply treatment (W0) received 623 mm of water from natural rainfall during the period of the experiment, which was higher than the mean rainfall of the previous 10 years for the area (456 mm) (Figure 2). The distribution was uneven during the experiment: 206 mm in November, 114 mm in December, 55 mm in January, 31 mm in February, 57 mm in March and 160 mm in April. For the even water treatment (W1), natural rainfall was excluded from the treatments by rain-out shelters (200 μm clear greenhouse polyethylene film, allowing around 95% of sun light irradiation) and we supplied a fixed amount of 46.3 mm (185 l per 2×2 m plots) of water to the seedlings every two weeks for the six months of the experiment, yielding a total of 556 mm water over the experiment, using sprinkler irrigation systems. Due to the lack of the rainfall data from the research site, the amount of water applied in W1 was based on the water deficit rules as defined in the Köppen-Geiger climate classification (550 mm per season), based on a recent update of these regional classifications [36].

To increase the nutrient availability for the tree seedlings in the high nutrient treatment (treatment N1), we used a granular slow-release inorganic fertilizer containing nitrogen (N), phosphorus (P) and potassium (K) in the ratio 3∶1∶2 (Osmocote Exact Standard 15∶9∶11, Scotts International, The Netherlands). The fertilizer was added once before the seedlings being transplanted in treatment seedlings at a rate of 4 g N m^−2^ (640 g per plot), following rates previously applied [15]. Normal annual amount of nitrogen mineralized in the study region was estimated at 5.8 g N m^−2^ [53], so N1 treatment increased local nitrogen availability ca.1.7 times.

### Shoot Foliar Nutrient Concentration and Biomass

The shoots of seedlings were harvested six months after planting in May 2010. These were oven-dried at 70°C for at least 48 h, and their dry weights were measured. To quantify the concentration of the elements N, P and K in leaves, the leaf material was digested with a mixture of H_2_SO_4_, Se and salicylic acid [54]. The concentrations of N and P in the leaves were measured with a Skalar San-plus auto-analyzer, and K was measured with an Atomic Absorption Spectrometer (AAS) from Varian (Palo Alto, CA, USA).

### Data Analysis

To test how the different treatments affected leaf nutrient concentrations and biomass production, we used general linear mixed models (GLMM), using maximum likelihood [55]. Water regime (W) and nutrient addition (N) and functional species group (FG) were included as fixed variables. To account for inter-specific variability, species was treated as a random factor in the model (Species, 8 levels), and plot within experimental block. As the inclusion of block and plot position did not significantly improve the model (all plots were very close to each other), these two random factors were dropped from the final model. The individual species analyses are provided in Table S1.

Mixed model analyses were conducted in R (R Development Core Team, 2013 - version 3.0.2) using the *lmer* function of the package lme4 [55]. To test the significance of the terms in the statistical model we ran Monte Carlo Markov Chain simulations (100,000 iterations) using the LanguageR package (http://cran.r-project.org/web/packages/languageR/languageR.pdf) to analyse the seedling biomass production and leaf nutrient concentrations.

The data used for this manuscript is made available via SANParks Data Repository website (https://knb.ecoinformatics.org/knb/style/skins/sanparks/index.jsp) and can also be obtained from the corresponding author.

## Results

**Figure 3:**
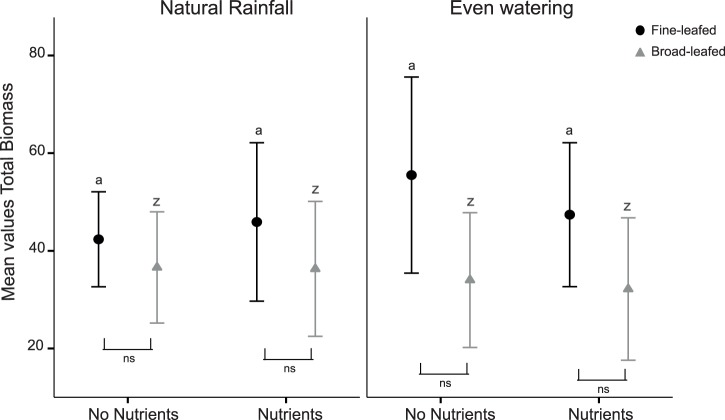
Effect of soil water and nutrient supply on the mean above-ground dry biomass (g). Bars represent 95% confidence intervals. The black circles represent the fine-leafed species, and the grey triangles represent the broad-leafed species. Nutrients represents the where Nitrogen-Phosphorus-Potassium (NPK) fertilazer was added, and No nutrients represents the plots where no NPK fertilazer was added. Statistical details are presented in Table 2. The letters represent differences between the treatments. Results of pairwise comparisons between the species groups within a treatment combination are indicated by the brackets (*: p<0.05; **: p<0.01; ***: p<0.001; ns: non-significant).

Contrary to our expectations (Hypothesis 1), short-term changes in soil resource availability (water and nutrient availability) did not affect above-ground biomass production of any of the tree species (Table 2 and Figure 3).

**Table 2:** The effect of functional species groups (FG) (fine-leafed vs. broad-leafed) of water (regular water supply vs. natural rainfall) and nutrient (no addition vs. NPK addition) treatments on leaf nutrient concentrations and above-ground biomass.

	Leaf nitrogen concentration			Leaf phosphorus concentration			Leaf potassium concentration			Total Biomass		
	Post mean	effective samples	p- MCMC	Post mean	effective samples	p- MCMC	Post mean	effective samples	p- MCMC	Post mean	effective samples	p- MCMC
FG	**−0.49**	**−0.14**	**0.011**	0.004	0.024	0.96	−0.01	0.20	0.93	−0.24	0.23	0.27
Nutrients	−**0.13**	−**0.01**	**0.029**	−**0.01**	−**0.003**	**0.015**	−0.03	0.05	0.44	−0.07	0.10	0.45
Water	−**0.24**	−**0.12**	**<0.0001**	−**0.01**	−**0.003**	**0.015**	−**0.10**	**0.00**	**0.040**	−0.07	0.10	0.46
FG × Nutrients	−0.09	0.09	0.31	−0.01	0.008	0.42	0.02	0.15	0.73	0.02	0.28	0.85
FG × Water	−0.01	0.17	0.92	0.005	0.020	0.49	−0.07	0.06	0.28	−0.07	0.18	0.58
Water × Nutrients	**0.20**	**0.37**	**0.024**	**0.03**	**0.045**	**<0.0001**	0.12	0.25	0.06	0.09	0.34	0.47
FG x Nutrients × Water	−0.07	0.17	0.60	0.01	0.026	0.56	−0.03	0.15	0.73	−0.08	0.26	0.68

Species was used as a random variable to correct for the variation among the different species. P values were obtained with Monte Carlo Markov Chain simulations (100000 iterations), using the MCMCglmm package and LanguageR package for R software (R Development Core Team, 2013, version 3.0.2). The significant values are represented in bold.

**Figure 4:**
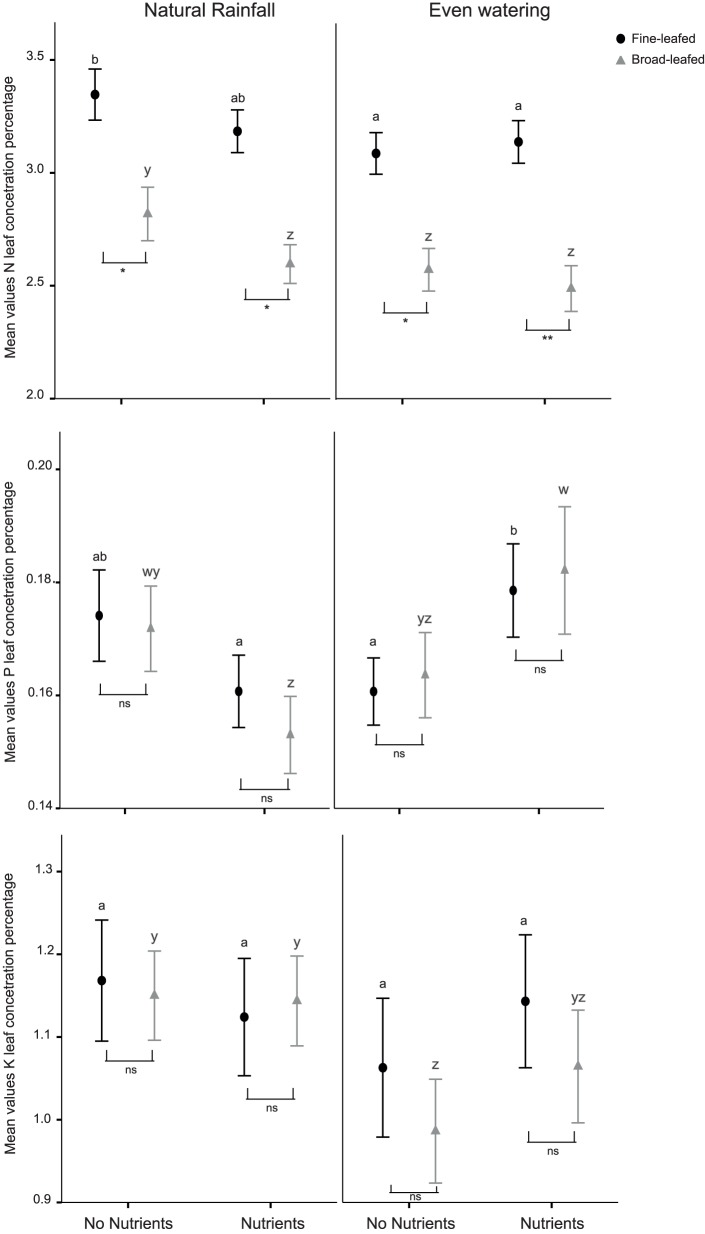
Effect of soil water and nutrient supply on the mean leaf nutrient concentration. Bars represent 95% confidence intervals. N = leaf nitrogen concentration, P = leaf phosphorus concentration and K = leaf potassium concentration. The black circles represent the fine-leafed species, and the grey triangles represent the broad-leafed species. “Nutrients” indicates where Nitrogen-Phosphorus-Potassium (NPK) fertilizer was added, and “No nutrients” represents the plots where no NPK fertilizer was added. Statistical details are presented in Table 2. The letters represent differences between the treatments. Results of pairwise comparisons between the species groups within a treatment combination are indicated by the brackets (*: p<0.05; **: p<0.01; ***: p<0.001; ns: non-significant).

In relation to leaf quality, we expected that fine-leafed species would present higher leaf nutrient concentrations than broad-leafed species (Hypothesis 2). Indeed, overall leaf N concentration was lower in broad-leafed species, but no significant differences were found between the two groups for leaf K and P concentration. In relation to the responses of leaf quality to treatments, we expected that leaf nutrient concentration would increase with increasing soil nutrient availability, and that it would decrease with constant water supply (Hypothesis 3). However, leaf P concentration only increased with NPK fertilizer input under even water supply in both species groups (Figure 4). A non-significant positive trend in leaf K concentration was also apparent. In contrast, under uneven water supply (natural rainfall), foliar concentrations of P and N were lower under the nutrient addition treatment (Table 2 and Figure 4). This trend was more accentuated for broad-leafed species with respect to leaf N concentration. Moreover, leaf K concentration of broad-leafed species was significantly lower than fine-leafed species when grown under constant water supply.

## Discussion

Plant productivity and above-ground biomass are thought to increase with higher soil resources (e.g. nitrogen, phosphorus) and water availability [8]–[10]. However, our results show that changes in leaf nutrient concentration varied with changes in the soil resource availability, even when biomass is not affected. Here we discuss the variability of responses to nutrient and water supply of two functional groups of tree species that are representative for the African savanna biome as a whole.

### Effect of Water and Nutrient Availability on Shoot Growth and Leaf Nutrient Concentration

Contrary to our expectations (Hypothesis 1), increased short-term nutrient input and water availability did not significantly influence above-ground biomass production of the two functional species groups studied. Three plausible explanations arise. Firstly, the nutrient additions may have been insufficient to cause a difference in growth between the unfertilised and fertilised plots (raised the available N in the soil by at most 70%). Indeed, as we used a slow release fertilizer, nutrients added may not have been immediately available. However, nutrient addition had a strong effect on leaf chemical composition, suggesting that nutrient additions did increase nutrient uptake by the tree seedlings. A second explanation is that seedlings of the considered species are not limited by soil nutrient availability in the study, savanna tree species being able to cope with low resource conditions. While our study included fine-leafed species, which can be found in regions with high nutrient soils (e.g. *Acacia* species), the seeds used in this study were collected in areas with relative low soil nutrients. It is, therefore, possible that the source populations of the seeds used in this study are adapted to grow in relatively infertile soils exhibiting lower maximum potential growth rates, and responding less to nutrient addition [56]. Moreover, under frequent water supply and high nutrient availability, it is possible that plant species of semi-arid environments allocate more resources to the root system [57] while above ground biomass remains constant.

The lack of growth response to improved fertility has been observed previously for tree seedlings growing in low nutrient environments [32], [58]. While the application of nutrient fertilizers may mitigate the adverse effects of water stress on plant development [59], the potential effect of nutrient addition on plants depends on their growth potential [58]. Therefore, changes in tree species composition are gradual, potentially taking a long time to be noticeable [60]. A longer term experiment (e.g. several years) would be essential to verify if nutrient enrichment and changes in water supply have delayed effects on savanna tree growth and biomass.

In relation to leaf-nutrient concentration, as expected fine-leafed species had higher leaf nutrient concentrations than broad-leafed species (Hypothesis 2). Fine leafed species are dominant on eutrophic soils [33]–[36], and hence are likely to be adapted to this high nutrient availability. Indeed all fine-leafed species studied here are able to fix atmospheric nitrogen [51], leading to a higher access to nitrogen. This extra N may be stored in leaves for future use, explaining the higher nitrogen values found in this study. Such storage might be important for shoot biomass recovery after intense fire or herbivory, as the N stored in the leaves not lost in the defoliation event can enhance shoot and leaf growth rates [27], which characterize nutrient rich savannas [35], [36]. Fine leafed species could, hence, show high growth rate [61]. Indeed, in all treatments the average biomass of the fine-leafed species was higher than for broad-leafed species, which could be due to this extra nitrogen availability. However, these differences between the two groups were not significant, which possibly due to the short period of the experiment.

In contrast to plant productivity, plant quality (measured as the nutrient concentration in leaves) did significantly vary with nutrient input and water availability (Figure 4). A previous study of leaf nutrient concentration of grasses [18] suggested that plant quality (i.e. leaf nutrient concentration) increases with soil nutrient concentration and decreases with water availability (similar to Hypothesis 3). Indeed, for both functional groups, leaf nitrogen concentrations were higher under natural conditions (natural rainfall/no NPK input) than in other treatments (Fig. 4). For plants growing under high N levels, plants may invest mostly in growth, leading to a dilution effect of N content of leaves [18], [50], potentially explaining the lower N content found in treatments with NPK addition. At low soil N availability, most N is stored in the leaves as amino acids, amides, or protein (enzymes such as Rubisco) [58]. The total Rubisco in the leaves increases linearly with increase of leaf N content, being essential for photosynthesis [62]. However, the activation of the Rubisco is regulated by CO_2_ levels in the leaves [63]. As leaf N content and CO_2_ assimilation by the leaves have a non-linear relationship, and hence most of the Rubisco in the leaves is inactive [64], not being used in photosynthesis. Such storage of N (e.g. as amino acids, or inactive Rubisco) can, however, be exported to support growth of other parts of the plant, whenever is needed [65].

In contrast with our expectations, our empirical results with savanna tree seedlings show that nutrient input increased leaf phosphorus concentration only when water input was regular, whereas decreases in leaf concentrations for this element occurred when water availability was uneven. Seedling dependence on water for a positive effect of nutrient (K and P) availability in leaves can be explained by the fact that nutrient uptake depends on water movement within plants [66]. Furthermore, the negative effect of nutrient input under the uneven water supply treatment (natural rainfall) also suggests that such irregularity in water provision stimulates allocation of resources away from leaves towards other organs, such as roots, that can support growth and survival when soil reserves are unavailable [67] for example during the dry (non-growing) season. This re-allocation is more likely to be noticeable at the end of the growing season, when our measurements were taken. Further studies on root production in tree seedlings across soil nutrient and moisture gradients would help to confirm where absorbed nutrients are allocated.

### Implications for Herbivore-plant Relationships

The availability of soil nutrients is influenced by herbivore density through dung and urine [7]. The results of this short-term experiment suggests that when combined with the natural (i.e. uneven) rainfall patterns, high soil nutrient availability may lead to a decrease of the leaf quality of tree seedlings as forage for herbivores (due to lower nutrient concentration in leaves, in this study mostly nitrogen and phosphorus), even when overall biomass does not change (Figure 3). Tree seedlings are a common food source for herbivores, especially due the high nutrient levels, low levels of defensive structures, and secondary defensive compounds [68]. This decline in leaf quality might, hence, increase the need of consumption by herbivores to acquire the amounts of nutrients needed by them, magnifying the effects of high herbivore density. This increase in browsing may affect tree recruitment, potentially impacting long-term dynamics and vegetation structure in savannas [22], [69]. Further changes in soil resource levels (e.g. higher N deposition, changes in wet season rainfall patterns) can lead to further accentuation of impacts of increased herbivore density for plant community dynamics in savannas. As several herbivore species are limited by the nutrient concentration of tree leaves, especially in pregnant and lactating animals [43], such changes may affect their population dynamics (e.g., reproduction, breeding times, and foraging range [14], [16], [18], [43]. Future studies would be needed to test such potential effects of soil nutrient input on herbivores, via change in tree seedlings nutrient content.

### Concluding Remarks

The results of our short-term multi-species experiment show that differences in soil resource availability lead to changes in leaf quality (leaf nutrient concentration). The effect of nutrient input on leaf quality (especially nitrogen and phosphorus concentrations) depends on water availability. Under more uneven water availability, leaf nutrient concentration decreases, while under regular rainfall it increases. While changes in the soil conditions might not directly affect plant species distribution [7], the changes in leaf quality may affect browsing pressures, and consequently affect overall vegetation structure. Our results hence suggest that, in response to the predicted changes in the rainfall distribution during the wet season in Southern African savannas (which is expected to become more erratic, with increases of the interval between each rainfall event [3], [41], leaf quality of tree seedlings for a large number of species will change, potentially affecting vegetation communities and herbivore population dynamics. Long-term experiments across multiple growing seasons are essential to confirm the robustness of the results obtained in this study. Moreover, close monitoring of how vegetation and herbivore communities will change in response to climate and land-use changes is essential both to understand the full extent of the ecological and consequences and to contributing to the development of adequate policies and management plans that aim to preserve biodiversity.

## Supporting Information

Table S1 **Effect of water (regular water supply vs. natural rainfall) and nutrient (no addition vs. NPK addition) treatments on leaf nutrient concentrations and above-ground biomass in all study species used in this research.** P values were obtained with Monte Carlo Markov Chain simulations (100000 iterations), using the MCMCglmm package and LanguageR package for R software (R Development Core Team, 2013).
(DOCX)
