# Supplementary material for: Short-Term Effect of Nutrient Availability and Rainfall Distribution on Biomass Production and Leaf Nutrient Content of Savanna Tree Species
Source: PLoS One. 2014 Mar 25;9(3):e92619. doi: 10.1371/journal.pone.0092619 (PMC3965441; doi:10.1371/journal.pone.0092619)
Supplement: Table S1 — Effect of water (regular water supply vs. natural rainfall) and nutrient (no addition vs. NPK addition) treatments on leaf nutrient concentrations and above-ground biomass in all study species used in this research. P values were obtained with Monte Carlo Markov Chain simulations (100000 iterations), using the MCMCglmm package and LanguageR package for R software (R Development Core Team, 2013). (DOCX) [file pone.0092619.s001.docx]

**Supplementary information**

**Table S1.** Effect of water (regular water supply vs. natural rainfall) and nutrient (no addition vs. NPK addition) treatments on leaf nutrient concentrations and above-ground biomass in all study species used in this research. P values were obtained with Monte Carlo Markov Chain simulations (100000 iterations), using the MCMCglmm package and LanguageR package for R software (R Development Core Team, 2013).

|  | Leaf nitrogen concentration | | | Leaf phosphorus concentration | | | Leaf potassium concentration | | | Total Biomass | | |
| --- | --- | --- | --- | --- | --- | --- | --- | --- | --- | --- | --- | --- |
|  | Post mean | effective samples | p- MCMC | Post mean | effective samples | p- MCMC | Post mean | effective samples | p- MCMC | Post mean | effective samples | p- MCMC |
| ***A. karroo*** |  |  |  |  |  |  |  |  |  |  |  |  |
| Water | 3.38 | 0.44 | 0.99 | -0.008 | 0.029 | 0.64 | -0.12 | 0.04 | 0.16 | 0.24 | 0.81 | 0.40 |
| Nutrients | 0.003 | 0.36 | 0.75 | 0.01 | 0.049 | 0.49 | -0.32 | -0.16 | **0.0004** | 0.12 | 0.73 | 0.66 |
| Water × Nutrients | -0.070 | 0.60 | 0.90 | 0.008 | 0.059 | 0.75 | 0.23 | 0.476 | **0.05** | -0.17 | 0.64 | 0.67 |
| ***A. nigrescens*** |  |  |  |  |  |  |  |  |  |  |  |  |
| Water | -0.05 | 0.14 | 0.57 | -0.01 | -0.001 | 0.029 | 0.01 | 0.18 | 0.89 | 0.010 | 0.33 | 0.94 |
| Nutrients | -0.26 | -0.07 | **0.006** | -0.01 | -0.003 | **0.011** | -0.18 | -0.009 | **0.03** | 0.14 | 0.45 | 0.36 |
| Water × Nutrients | 0.06 | 0.33 | 0.66 | 0.03 | 0.05 | 0.004 | 0.13 | 0.38 | 0.26 | -0.07 | 0.36 | 0.73 |
| ***A. nilotica*** |  |  |  |  |  |  |  |  |  |  |  |  |
| Water | 0.02 | 0.26 | 0.87 | 0.003 | 0.024 | 0.74 | -0.08 | 0.12 | 0.42 | -0.06 | 0.34 | 0.77 |
| Nutrients | -0.02 | 0.21 | 0.80 | -0.0009 | 0.020 | 0.93 | 0.05 | 0.26 | 0.60 | -0.03 | 0.39 | 0.86 |
| Water × Nutrients | -0.15 | 0.18 | 0.36 | -0.008 | 0.021 | 0.54 | 0.07 | 0.39 | 0.61 | 0.09 | 0.71 | 0.74 |
| ***A. sieberiana*** |  |  |  |  |  |  |  |  |  |  |  |  |
| Water | -0.35 | -0.08 | **0.015** | -0.01 | 0.008 | 0.20 | -0.13 | 0.03 | 0.11 | -0.42 | 0.03 | **0.05** |
| Nutrients | -0.29 | -0.0085 | **0.044** | -0.007 | 0.012 | 0.51 | -0.15 | 0.01 | 0.09 | 0.11 | 0.55 | 0.61 |
| Water × Nutrients | 0.59 | 1.0107 | **0.006** | 0.05 | 0.090 | **0.001** | 0.16 | 0.41 | 0.18 | 0.35 | 0.99 | 0.26 |
| ***A. tortilis*** |  |  |  |  |  |  |  |  |  |  |  |  |
| Water | -0.36 | -0.07 | **0.016** | -0.021 | 0.0009 | **0.05** | -0.08 | 0.13 | 0.42 | 0.16 | 0.61 | 0.44 |
| Nutrients | -0.43 | -0.14 | **0.004** | -0.032 | -0.01 | **0.003** | -0.33 | -0.12 | **0.00**3 | 0.040 | 0.45 | 0.85 |
| Water × Nutrients | 0.60 | 1.05 | **0.006** | 0.057 | 0.09 | **0.0008** | 0.28 | 0.59 | 0.07 | -0.060 | 0.58 | 0.85 |
| ***C. apiculatum*** |  |  |  |  |  |  |  |  |  |  |  |  |
| Water | -0.15 | 0.13 | 0.29 | -0.03 | -0.0005 | **0.036** | -0.05 | 0.11 | 0.51 | -0.27 | 0.03 | 0.07 |
| Nutrients | -0.25 | 0.04 | 0.09 | -0.01 | 0.019 | 0.34 | -0.37 | -0.20 | **0.0002** | -0.27 | 0.01 | 0.06 |
| Water × Nutrients | 0.09 | 0.50 | 0.65 | 0.07 | 0.125 | **0.0016** | 0.17 | 0.41 | 0.16 | 0.108 | 0.51 | 0.59 |
| ***C. mopane*** |  |  |  |  |  |  |  |  |  |  |  |  |
| Water | -0.04 | 0.12 | 0.60 | -0.0001 | 0.014 | 0.99 | 0.011 | 0.15 | 0.86 | 0.13 | 0.58 | 0.57 |
| Nutrients | -0.35 | -0.19 | **0.0001** | -0.01 | 0.0018 | **0.07** | -0.13 | -0.003 | **0.047** | -0.044 | 0.42 | 0.84 |
| Water × Nutrients | 0.23 | 0.47 | **0.046** | 0.018 | 0.039 | 0.09 | 0.14 | 0.34 | 0.15 | -0.090 | 0.58 | 0.79 |
| ***D. cinerea*** |  |  |  |  |  |  |  |  |  |  |  |  |
| Water | -0.08 | 0.43 | 0.42 | -0.009 | 0.009 | 0.32 | 0.05 | 0.61 | 0.62 | -0.39 | -0.10 | **0.009** |
| Nutrients | -0.14 | 0.15 | 0.15 | 0.007 | 0.026 | 0.44 | 0.11 | 0.29 | 0.29 | -0.44 | -0.15 | **0.003** |
| Water × Nutrients | 0.20 | 0.14 | 0.14 | 0.03 | 0.056 | **0.03** | -0.06 | 0.68 | 0.69 | 0.45 | 0.86 | **0.023** |
| ***P. africanum*** |  |  |  |  |  |  |  |  |  |  |  |  |
| Water | -0.18 | 0.05 | 0.14 | -0.02 | -0.0032 | **0.021** | -0.013 | 0.13 | 0.85 | 0.036 | 0.27 | 0.75 |
| Nutrients | -0.04 | 0.19 | 0.72 | 0.002 | 0.019 | 0.76 | -0.14 | 0.0009 | **0.053** | -0.24 | 0.001 | **0.047** |
| Water × Nutrients | 0.04 | 0.40 | 0.80 | 0.019 | 0.042 | 0.10 | 0.020 | 0.2 | 0.84 | 0.11 | 0.43 | 0.50 |
| ***S. brachypetala*** |  |  |  |  |  |  |  |  |  |  |  |  |
| Water | -0.52 | -0.22 | **0.001** | -0.02 | -0.0019 | **0.037** | -0.009 | 0.14 | 0.89 | -0.14 | 0.12 | 0.32 |
| Nutrients | -0.3 | -0.002 | **0.047** | -0.005 | 0.012 | 0.55 | -0.036 | 0.11 | 0.64 | 0.019 | 0.30 | 0.90 |
| Water × Nutrients | 0.13 | 0.56 | 0.54 | 0.03 | 0.06 | **0.010** | 0.047 | 0.25 | 0.65 | -0.030 | 0.38 | 0.89 |
